# Review on the Recent Advances on Typhoid Vaccine Development and Challenges Ahead

**DOI:** 10.1093/cid/ciaa504

**Published:** 2020-07-29

**Authors:** Khalid Ali Syed, Tarun Saluja, Heeyoun Cho, Amber Hsiao, Hanif Shaikh, T Anh Wartel, Vittal Mogasale, Julia Lynch, Jerome H Kim, Jean-Louis Excler, Sushant Sahastrabuddhe

**Affiliations:** 1 MSD-Wellcome Trust Hilleman Laboratories Pvt Ltd, New Delhi, India; 2 International Vaccine Institute, Seoul, Republic of Korea; 3 Technische Universität Berlin, Berlin, Germany; 4 KEM Hospital Research Center, Pune, India

**Keywords:** typhoid fever, *Salmonella* typhi, typhoid conjugate vaccine, Vi-polysaccharide vaccine, nontyphoidal *Salmonella*

## Abstract

Control of *Salmonella enterica* serovar typhi (*S.* typhi), the agent of typhoid fever, continues to be a challenge in many low- and middle-income countries. The major transmission route of *S*. typhi is fecal-oral, through contaminated food and water; thus, the ultimate measures for typhoid fever prevention and control include the provision of safe water, improved sanitation, and hygiene. Considering the increasing evidence of the global burden of typhoid, particularly among young children, and the long-term horizon for sustained, effective water and sanitation improvements in low-income settings, a growing consensus is to emphasize preventive vaccination. This review provides an overview of the licensed typhoid vaccines and vaccine candidates under development, and the challenges ahead for introduction.

Typhoid fever, an invasive bacterial infection caused by *Salmonella enterica* serovar typhi (*S.* typhi), remains an important public health threat worldwide, particularly in low- and middle-income countries with poor access to safe water supplies and sanitation. Susceptible human hosts usually ingest *S.* typhi through contaminated food or water [1]. Inside the small intestine, *S.* typhi attaches to intestinal cells and, after penetrating the epithelium into the *lamina propria*, the organisms disseminate into the bloodstream in a primary bacteremia that seeds the reticuloendothelial system, which allows *S.* typhi to reside for a relatively long period in organs before inducing a range of clinical illness. The incubation period is usually 8–14 days [2]. During the primary bacteremia following ingestion of typhoid bacilli and seeding of the reticuloendothelial system, organisms also reach the gallbladder, an organ for which *S.* typhi has a preferred tropism [3] and which is linked with the long-term carrier status.

The real disease burden of typhoid fever worldwide is not well defined due to the lack of surveillance efforts in many areas, the heterogeneity of the disease presentation, and the difficulty in confirming the diagnosis [4]. Various modeling studies have estimated that the disease burden ranges from 12 million to 21 million cases per year and 129 000 to 145 000 deaths annually worldwide [5–7]. The disease burden is high in low- and middle-income countries, particularly in Asia [8, 9] and sub-Saharan Africa [10, 11] and is mostly concentrated in areas with poor hygiene and sanitation, like urban slums and rural areas without access to clean water [12]. A challenge in managing enteric fever is growing antimicrobial resistance; since the first reports of chloramphenicol resistance in *S.* typhi in the 1970s, resistance to each new antimicrobial treatment has emerged relentlessly [13]. Multidrug resistance—that is, resistance to chloramphenicol, amoxicillin, and co-trimoxazole—is found in many areas of South Asia and was associated with numerous outbreaks in the late 1980s and early 1990s [14].

The recent emergence of extensively drug resistant *S*. typhi strains resistant to fluoroquinolones and third-generation cephalosporins, in addition to first-generation antibiotics, has made the treatment of typhoid fever difficult and expensive [15]. With increasing evidence of the burden of typhoid fever in younger children, particularly those <2 years of age, early programmatic intervention with vaccination is critical [16].

The long-term and proven strategies for typhoid prevention and control are the provision of safe drinking water, development of sanitary infrastructure, and implementation of hygienic practices. However, the development of infrastructure for safe water and sanitation requires substantial and long-term investments, which may take decades to realize in low- to middle-income countries. As an intermediate measure, basic health education in handwashing and improved food handling, along with the use of an effective vaccine, represent reasonable and effective tools.

Earlier typhoid vaccines have been recommended for use in adults and children >2 years of age. The live attenuated Ty21a vaccine was licensed in Europe in 1983 and the United States in 1989; the Vi-polysaccharide vaccine was first licensed in the United States in 1994 [17]. Although efficacious, these vaccines have several limitations precluding their wider use in endemic countries and in children <2 years of age (see below).

To that end, Typbar TCV, a typhoid conjugate vaccine (TCV), was prequalified by the World Health Organization (WHO) in January 2018. Typbar TCV is manufactured by Bharat Biotech International Limited and is indicated for use in individuals aged 6 months to 45 years. Subsequently, the WHO Strategic Advisory Group of Experts (SAGE) has recommended that the introduction of TCV be prioritized in countries with the highest burden of typhoid disease or a high burden of antimicrobial resistance to *S.* typhi [18]. The WHO recommends routine use of TCV, along with other vaccines, at 9 months of age or in the second year of life, as necessitated by the local situation in endemic countries. The introduction of TCV through routine immunization is among the most effective interventions for the youngest age groups. Depending on vaccination strategies and the speed of country adoption, the forecasted annual demand of TCV may increase up to 160 million doses under the rapid introduction scenario [19]. Even with a WHO prequalification of 1 TCV (Typbar TCV), there will still be an unmet need of TCV in the global public market. In this review, we will present the current developmental status of various TCV candidates, along with other typhoid vaccines.

## FIRST-GENERATION TYPHOID VACCINES

Heat- and phenol-inactivated whole-cell vaccines against typhoid have been available since the late 19th century. Large-scale use of these vaccines in British and American soldiers resulted in a considerable reduction in the typhoid fever incidence. In the 1960s and 1970s, controlled field trials were conducted in British Guyana, Tonga, the Union of Soviet Socialist Republics, and Egypt to study the efficacy of these vaccines. Studies indicated that the vaccines had an efficacy of 51–88% against typhoid fever and that protection lasted up to 7 years [20]. However, the high frequency of reactogenicity (fever, headache, and pain at injection site) in vaccine recipients led to the withdrawal of these vaccines from routine immunization programs [21].

## SECOND-GENERATION TYPHOID VACCINES

Since the late 1980s, 2 types of second-generation vaccines have been licensed for use: an oral live attenuated vaccine and an injectable subunit Vi-capsular polysaccharide vaccine (Table 1).

**Table 1.** Characteristics of the 2 Typhoid Vaccines Currently Recommended by the World Health Organization: Ty21a and Vi Polysaccharide

	Ty21a Vaccine	Vi Capsular Polysaccharide Vaccine
Vaccine type	Live attenuated	Subunit
Composition	Chemically mutated Ty2 strain of *S.* typhi	Purified Vi capsular polysaccharide of Ty2 *S.* typhi strain
Immunogenic properties	▪Elicits mucosal IgA and serum IgG antibodies against O, H, and other antigens, as well as cell-mediated responses ▪No booster effect has been shown	▪Elicits serum IgG Vi antibodies ▪T-cell independent (no booster response)
Route of administration	Oral	Parenteral (subcutaneous or intramuscular)
Minimum age vaccine is licensed for use	2 years old for liquid formulation and 5 years old for capsule formulation	2 years old
Formulation	▪Enteric-coated capsules, or ▪Liquid suspension (lyophilized vaccine + buffer mixed with water upon use)	Solution of 25 µg combined with buffer
Number of doses required for complete vaccine regimen	3 to 4	1
Storage requirements	Requires storage at 2º to 8ºC	Requires storage at 2º to 8ºC
Shelf life in higher temperature	14 days at 25 °C	6 months at 37 °C 2 years at 22 °C
Safety/tolerability	High	High
Efficacy at 3 years (95% CI)	51% (36–62%)	55% (30–70%)
Length of protection	At least 5–7 years	At least 3 years

Abbreviations: CI, confidence interval; Ig, immunoglobin; *S.* typhi, *Salmonella enterica* serovar typhi.

### Live Attenuated Vaccine

The live attenuated Ty21a typhoid vaccine was developed by chemical mutagenesis of the Ty2 *S.* typhi strain. In large-scale efficacy studies conducted in Chile, Egypt, and Indonesia, the protective efficacy of 3 doses was 42–96% (Table 2) [22, 23]. In clinical trials, 2 formulations were developed and tested: an enteric-coated capsule (for use in individuals ≥5 years of age) and a liquid formulation (lyophilized liquid reconstituted in buffer, for use in individuals ≥2 years of age). Both types of vaccines were well tolerated and protection after 3 doses lasted up to 7 years [22]. Currently only 1 formulation is commercially available in the form of a capsule for use in individuals ≥5 years of age.

**Table 2.** Summary of Studies Undertaken for the Ty21a Vaccine in Developing Countries

Study (Year)	Formulation	No. Study Subjects	Ages, Years	Follow-up Period	Protective Efficacy for Blood Culture Confirmed Typhoid (95% CIs)	Incidence Rate in Control Group, per 100 000
Alexandria, Egypt (1978–1980)	Liquid given with tablet of NaHCO3	32 388	6–7	36 months	96% (77–99%)	50
Area Occidente, Santiago, Chile (1983–1986)	3 doses of enteric-coated capsules given (1–2 days between doses)	140 000	6–19	36 months 7 years	67% (47–79%) 62%	110
Area Sur Oriente, Santiago, Chile (1986)	3 doses of enteric-coated capsules (1–2 days between doses)	81 321	6–19	3 years	33% (0–57%)	100
	3 doses liquid suspension (1–2 days between doses)			3 years 5 years	77% 78%	
Sumatra, Indonesia (1986–1989)	3 doses of enteric-coated capsules (7 days between doses)	20 543	3–44	30 months	42% (23–57%)	810
	3 doses liquid suspension (7 days between doses)				53% (36–66%)	

Abbreviation: CI, confidence interval.

### Capsular Polysaccharide Vaccine

A subunit vaccine consisting of a purified Vi capsular polysaccharide of *S.* typhi strain Ty2 was developed by the US National Institutes of Health (NIH) [24]. A similar injectable formulation of the Vi-polysaccharide was also developed by Sanofi Pasteur, which elicited an anti-Vi antibody response in 85–95% of individuals >2 years of age with a single dose [17]. The vaccine efficacy for *S.* typhi was 64–72% for 17–21 months and 55% over a period of 3 years [25].

Vi-polysaccharide vaccines have been produced by several manufacturers from developed countries, as well as developing countries. They have been widely used in various settings and in routine immunization programs [26]. Only 1 Vi-polysaccharide vaccine (produced by Sanofi Pasteur) is prequalified by the WHO. Common side effects associated with this vaccine include pain, redness, injection site induration, and fever. Rare allergic reactions and rashes have been observed following vaccination. This vaccine (like other polysaccharide vaccines) is not immunogenic in children <2 years of age and is only licensed for use in individuals ≥2 years of age. Owing to the short duration of protective immunity, revaccination is advised every 3 years [17].

### Need for Better Typhoid Vaccines

Even though both live attenuated Ty21a and Vi-polysaccharide vaccines are effective, several limitations hamper their inclusion in the Expanded Programme on Immunizations (EPI) schedules of typhoid-endemic countries. Live attenuated vaccines are available in capsule formulation only and, hence, cannot be administered to children <5 years of age. Multiple doses are required to complete a vaccination course, and revaccination is recommended every 5 years. This vaccine also requires a strict cold chain to be maintained during storage and handling, which is a major limiting factor in resource-poor settings [17].

For the Vi vaccines, as with other polysaccharide vaccines, including pneumococcal and meningococcal vaccines [27], more fundamental immunological limitations preclude their widespread use. The Vi vaccine–induced immune response is elicited by a T cell–independent mechanism, to which children <2 years of age respond poorly. Further, there is no development of immune memory. As a consequence, only short-term responses are generated and there is no boosting following a second vaccination that would lead to a shorter duration of protection. Revaccination is necessary every 3 years for children >2 years [28]. Most manufacturers are abandoning Vi-polysaccharide vaccines in favor of TCV, and the product is no longer available in most endemic countries.

### Live Oral Vaccines

Several groups are working to improve the immunogenicity of the live attenuated vaccine and reduce the number of doses needed for effective immunization by using advanced molecular technology [29]. One vaccine candidate uses the *S.* typhi Ty2 strain with deletion in the PhoP/PhoQ genes, which results in the deactivation of PhoP/PhoQ regulon. PhoP/PhoQ regulon governs virulence and regulates a number of other cellular activities in *Salmonella* [30, 31].

This vaccine, Ty 800, was tested in a dose-escalation study in 11 human volunteers [32]. Another live attenuated vaccine candidate, Center for Vaccine development, University of Maryland (CVD) 908-htrA, was developed by deleting the aroC/aroD and htrA gene locus. CVD 908-htrA was first tested in 13 human volunteers, followed by a Phase II study in 80 participants in the United States [33]. Investigators at the University of Maryland mutated the aroC and ssaV genes and tested vaccine candidate M01ZH09 in 32 human volunteers, followed by larger studies in the United States and Vietnam [34, 35]. All 3 vaccine candidates—Ty800, CVD908-htrA, and M01ZH09—were safe and induced significant immune responses after a single dose. The M01ZH09 vaccine was recently tested in a human challenge study at Oxford University in the United Kingdom. A single dose of M01ZH09 failed to demonstrate significant protection after a challenge with virulent *S.* typhi [36].

Another vaccine candidate, CVD 909, was tested with the Vi-polysaccharide vaccine in a prime-boost regimen, with CVD 909 given orally followed by an injection of Vi-polysaccharide vaccine 3 weeks later. Priming with CVD 909 elicited higher and more persistent, albeit not significant, anti-Vi immunoglobin (Ig) G and A following immunization with Vi than priming with a placebo. Vi-specific IgA B memory cells were significantly increased in CVD 909–primed subjects [37]. Though immunogenic, there will be a need for the preadministration of buffer to neutralize stomach acid with live attenuated vaccines, which is a potential delivery challenge.

### Improvements of Vi-Polysaccharide Vaccine

The strategy of conjugating the polysaccharide to a carrier protein has been successfully used for pneumococcal, meningococcal, and Haemophilus influenzae type B vaccine polysaccharide vaccines to overcome the limitations of polysaccharides alone as immunogens [38]. Conjugation to a carrier protein changes the antigenic property of the polysaccharide and makes it a T cell–dependent antigen. These antigens are immunogenic in younger children and infants, elicit a booster response to subsequent immunization, and have a longer duration of protection.

## TYPHOID CONJUGATE VACCINES 

Several groups have developed TCVs using Vi-polysaccharide from different sources and using different carrier proteins for conjugation. So far, 3 TCVs have been licensed, all in India, and are being distributed in the Indian private market, with 1 (Typbar-TCV) prequalified by the WHO and available for use for global public health. Multiple other TCV candidates are in various stages of development (Table 3).

**Table 3.** Current Typhoid Conjugate Vaccine Development Pipeline

Manufacturer	Location	Technology Transfer Agreement	Product Details	Clinical Development Status	WHO Prequalification
Bharat Biotech Int. Ltd	India	Own R&D	Vi-TT	Licensure in India, Nepal, Nigeria	WHO prequalified January 2018
Bio-Med Pvt. Ltd	India	Own R&D	Vi-TT	Licensure in India	No plans for WHO PQ as of now
M/s Cadila Healthcare Limited	India	Unknown	Vi-TT	Licensed in India March 2018	WHO PQ will be sought
PT Bio Farma	Indonesia	IVI	Vi-DT	Phase II	WHO PQ will be sought after Indonesian NRA
Finlay Institute	Cuba	Unknown	Vi-DT	Phase I	Unknown plans for WHO PQ
Lanzhou Institute (CNBG)	China	US NIH	Vi-rEPA	Licensure application submitted	Interest in WHO PQ; need support
SK Bioscience	S. Korea	IVI	Vi-DT	Phase II	WHO PQ will be sought after licensure
Incepta	Bangladesh	IVI	Vi-DT	Preclinical	Interest in WHO PQ; need support
Biological E	India	NVGH (GSK)	Vi-CRM	Phase III	WHO PQ will be sought after licensure
DAVAC	Vietnam	Own R&D	Vi-DT	Preclinical	NA
Eubiologics	Korea	Own R&D	Vi-TT	Phase I	Interest in WHO PQ: Unknown

Abbreviations: CNBG, China National Biotec Group; DT, diphtheria toxoid; IVI, International Vaccine Institute; NA, not available; NIH, National Institutes of Health; NVGH, Novartis Vaccines Institute for Global Health; NRA, National Regulatory agency; R&D, research and development; rEPA, recombinant A subunit of *Pseudomonas aeruginosa* exoprotein; TT, tetanus toxoid; WHO, World Health Organization.

### Vi-rEPA

Vi-rEPA, 1 of the earlier TCV candidates, was developed by the US NIH [35]. The *S.* typhi Vi-polysaccharide was conjugated to the recombinant A subunit of *Pseudomonas aeruginosa* exoprotein (rEPA). The conjugate was synthesized using adipic acid dihydrazide (ADH) as a linker to bind the Vi to the rEPA protein. Briefly, rEPA was prederivatized with the homo bifunctional linker ADH, and the Vi-polysaccharide was then covalently linked to derivatized rEPA in the presence of 1-ethyl-3-(3-dimethylaminopropyl) carbodiimide. The process was optimized to yield consistent and high levels of conjugation. In contrast to Vi-polysaccharide, Vi-rEPA elicited much higher levels of anti-Vi IgG in mice with a booster response to reinjection, suggesting a T cell–dependent mechanism of immune system activation. This process was adapted by other groups to produce Vi conjugates using other carrier proteins, like CRM_197_, diphtheria toxoid (DT), and tetanus toxoid (TT) [39].

A Vi-rEPA formulation with 25 μg of Vi-polysaccharide was developed and tested in clinical trials. The vaccine was well tolerated in an initial adult trial in Vietnam [40]. Anti-Vi antibody titers in vaccinated participants rose 50-fold at 1 month after immunization with Vi-rEPA. At 6 months following immunization, antibody titers were found to be 12-fold higher than baseline [40]. A Phase II study was conducted in children 5–14 years of age who were randomized to receive 1 dose of either the Vi-rEPA or Vi-polysaccharide vaccine. The anti-Vi antibody geometric mean titer (GMT) was significantly higher in Vi-rEPA recipients than in Vi-polysaccharide recipients. In the second part of the Phase II study, participants 2–4 years of age were randomized to receive 1 or 2 doses (6 weeks apart) of Vi-rEPA. After a single dose, 99.5% of participants had a more than 8-fold increase in anti-Vi IgG titers. The antibody titers of participants who received a second dose rose from 69.9 to 95.4 Elisa units (EU) at 4 weeks after the second dose. The difference in titers following 1 dose and 2 doses of Vi-rEPA was narrow (20.4 and 30.6 EU, respectively) at 26 weeks [40].

A randomized controlled trial to assess the efficacy of 2 doses of Vi-rEPA was conducted in children 2–5 years of age in Vietnam. There were 13 776 children, aged 2 to 5 years old, enrolled from 16 communes in Dong Thap Province, Vietnam. A total of 12 008 children received at least 1 injection, and 11 091 children received 2 injections of vaccine or placebo 6 weeks apart. During the 27-week follow-up period, there were 4 cases of typhoid fever in the vaccine group and 47 in children in the placebo group. The efficacy of two 25 μg doses given 6 weeks apart was found to be 91.5% (95% CI, 77.1–96.6) [41]. Further studies to study the safety and immunogenicity of various dosages (5, 12.5, and 25 μg) of Vi-rEPA and the persistence of antibodies were conducted. In Vietnam, 3 dosage strengths (5, 12.5, and 25 μg) were evaluated in 2- to 5-year-old children, and all 3 formulations resulted in more than 8-fold increase in titers at 10 weeks after administration of 2 doses. The immune responses were dose-dependent, with 25 μg eliciting the greatest response (102 EU/mL, 74.7 EU/mL, and 43 EU/mL for 25 μg, 12.5 μg, and 5 μg, respectively). This difference in the titers was statistically significant (*P* < .004). Also, the antibody titers at 1 year after the first injection were 6.43 EU/ml, 11.3 EU/ml, and 13.3 EU/ml for 5 μg, 12.5 μg, and 25 μg dose recipients, respectively [42].

A study to assess the safety, immunogenicity, and compatibility of Vi-rEPA with EPI vaccines was further conducted in Vietnamese infants. Doses of Vi-rEPA were given at 2, 4, 6, and 12 months of age. The results indicated that the vaccine was safe and immunogenic in infants and compatible with EPI vaccines. Vi-rEPA did not show interference in immune responses to coadministered, routinely used childhood vaccines, indicating that Vi-rEPA can be added to the routine immunization program [24].

The technology of this candidate vaccine was transferred to the Lanzhou Institute of Biological Products in China for further development. The Vi-rEPA is manufactured by the Lanzhou Institute, and randomized clinical trials were conducted. Results from the clinical trials were similar to the results of studies conducted by the NIH in Vietnam. Lanzhou is working with China’s National Medical Products Administration (the former China Food and Drug Administration) for licensure of this product [43].

### Vi-CRM_197_

Vi-CRM_197_ was developed by the Novartis Vaccines Institute for Global Health, Siena, Italy (now known as the GSK Vaccine Institute for Global Health). Vi-polysaccharide from *Citrobacter freundii* WR7011 was conjugated to CRM_197_, a nontoxic mutant of diphtheria toxoid [44]. A Phase I study was completed using a single 25 μg dose of Vi-CRM_197_ in healthy adults, with a Vi-polysaccharide vaccine (Typherix, GlaxoSmithKline; 0.5mL containing 25 μg Vi-polysaccharide) as a comparator. The GMTs 28 days after vaccination were 304 and 52 in Vi-CRM197 and Vi-polysaccharide recipients, respectively. At 6 months postvaccination, the difference in antibody titers of Vi-polysaccharide and Vi-CRM_197_ recipients narrowed to 51 and 69, respectively, suggesting a faster decline in the antibody titers of participants who received Vi-CRM_197_. A Phase II randomized dose-ranging study was conducted in 88 participants who received either 1.25 μg, 5 μg, or 12.5 μg of Vi-CRM_197_ or Vi-polysaccharide vaccine. The different formulations of the Vi-CRM_197_ were found to be safe. Anti-Vi antibody responses 4 weeks after vaccination were higher in participants receiving Vi-CRM_197_ (all 3 dosages) than in Vi-polysaccharide recipients. The response was dose-dependent, with the lowest titers found in participants receiving the 1.25 μg dose [45].

A multicenter, age deescalating, Phase II study was conducted in Pakistan, India, and the Philippines. The 5 μg formulation of Vi-CRM_197_ was tested in this study, and the Vi-polysaccharide vaccine was used as the comparator in all age groups except in children <2 years of age, where the pneumococcal conjugate vaccine was used as the control. Vi-CRM_197_ was found to be safe in all age groups and did not interfere with EPI vaccines. The immune response after a single dose of Vi-CRM_197_ in adults was higher than the single dose of the Vi-polysaccharide vaccine. In children, the response to the first dose of Vi-CRM_197_ was higher than that of the Vi-polysaccharide, but the second dose did not result in an increase in anti-Vi antibody titers. Further, at 6 months postvaccination, the anti-Vi antibody titers of recipients of either Vi-CRM_197_ or Vi-polysaccharide vaccines were similar, indicating a rapid decline in the titers of Vi-CRM_197_ recipients. The absence of a booster response to the second dose and the rapid decline in the anti-Vi antibody titer led to further process development and the improved technology was transferred to Biological E (Hyderabad, India) for further development and commercialization. A first-in-human clinical trial to assess the safety of Vi-CRM_197_ was conducted, followed by a Phase II/III study. The objective of the Phase II/III study was to demonstrate the noninferiority of immunogenicity of Biological E’s TCV to the licensed comparator (Typbar-TCV) in participants ≥6 months to <64 years of age [46–48]. Vi-CRM_197_ is expected to be licensed for use by Drugs Controller General of India in India in coming months.

### Vi-DT

The TCV candidate developed by the International Vaccine Institute, Seoul, Republic of Korea, consists of Vi-polysaccharide conjugated to DT (Vi-DT). The Vi-polysaccharide is harvested from the *S.* typhi strain C6524, a clinical isolate from India. The Vi-DT conjugate was synthesized using ADH as a linker to bind the Vi polysaccharide to the carrier protein DT. In animal experiments, Vi-DT was more immunogenic than Vi-polysaccharide alone [49]. The technology was initially transferred to Shantha Biotechnics in India in 2009, but in early in 2014 Sanofi, which had acquired Shantha, decided to discontinue the development of Vi-DT. The same technology had been transferred to 3 additional vaccine manufacturers: SK Bioscience in the Republic of Korea, PT Bio Farma in Indonesia, and Incepta Vaccines in Bangladesh.

SK Bioscience’s Vi-DT vaccine underwent preclinical evaluation in 2015 and clinical trial lots were prepared the same year. A randomized, observer-blinded, age deescalating, Phase I safety and immunogenicity study of SK Bioscience’s Vi-DT vaccine was completed in Manila, the Philippines. Vi-DT contains 25 µg of purified Vi-polysaccharide (*S.* typhi C6524) conjugated to DT formulated as 0.5 mL/vial. The study was conducted in an age deescalating manner in participants 18–45, 6–17, and 2–5 years of age, randomized to receive either 2 doses of Vi-DT or the comparator vaccines (Typhim-Vi and Vaxigrip, Sanofi Pasteur). Vi-DT was well tolerated in all age groups, most of the adverse reactions were mild or moderate in intensity, and pain at the injection site was the most common immediate reaction. No serious adverse events (SAE) were reported from this study. All participants (100%) in the Vi-DT group seroconverted by 28 days after the first dose and remained so on Day 56 after the second dose, while 97% of participants seroconverted in the comparator (Typhim-Vi) group. Vi-DT recipients had 4-fold higher GMTs compared with the comparator vaccine recipients [50]. A Phase II study with 2 years of long-term follow-up is going on in the Philippines, and preliminary results from the study are available. A total of 285 participants were enrolled and age-stratified: 6 to <9 months, 9–12 months, and 13–23 months. Per age strata, 76 participants received Vi-DT and 19 received placebo. All participants seroconverted after a single dose of Vi-DT, versus 7% of placebo recipients. The anti-Vi IgG GMT was 444.38 (95% confidence interval [CI], 400.28–493.34) after a single dose of Vi-DT; there was no change in the GMT after placebo administration (0.41; 95% CI, .33–.51; *P* < .0001). A similar pattern of immunogenicity was reported across all age strata. A single, unrelated SAE with the diagnosis of febrile convulsion secondary to a urinary tract infection was reported 5 days after Vi-DT administration in the 6- to <9-month age stratum. It was mild in severity and resolved without sequelae after medical management. No SAEs were reported from the placebo group. All unsolicited adverse events reported were of mild to moderate severity, and most of them were assessed as unrelated to Vi-DT or placebo administration. There was no statistical difference in the proportions of participants who experienced solicited adverse events within 7 days between Vi-DT and placebo groups [51].

PT Bio Farma has also completed a Phase I study in Jakarta, Indonesia. PT Bio Farma’s Vi-DT vaccine was also safe and immunogenic in participants >2 years of age [52]. A Phase II study is ongoing, including children 6–23 months of age [53]. The second dose of Vi-DT did not result in any significant increases in GMTs of anti-Vi antibodies in either of these studies, indicating that this could be a single-dose vaccine.

## LICENSED TYPHOID CONJUGATE VACCINES 

### PedaTyph From Biomed India

PedaTyph (a Vi-TT conjugated vaccine), manufactured by Biomed India, was the first licensed TCV in India. Each dose (0.5 mL) contains 5 µg of Vi-polysaccharide of *S.* typhi (strain Ty2), conjugated to tetanus toxoid protein. The vaccine was tested in a clinical study in 169 subjects >12 weeks of age, with a comparator group of 37 children >2 years of age who received the Vi-polysaccharide vaccine. The results of this study indicated that the vaccine was immunogenic in more than 90% participants. The results were posted on the company website, and PedaTyph was licensed for use in children >3 months of age. Initially, this vaccine came under some criticism due to a lack of sufficient data to support widespread use. The results of the licensure trial were subsequently published [54].

After a licensure based on immunogenicity only, a cluster-randomized clinical efficacy study was conducted in infants 6 months to 12 years of age in Kolkata, India. Out of 1765 participants enrolled in the study, 905 participants received PedaTyph and 860 were assigned to the placebo arm. The vaccine was well tolerated in this age group, with no reports of vaccine-related SAEs. Participants were followed up actively by weekly phone calls and monthly school visits to determine efficacy. The vaccine efficacy at 12 months was 100%, as no typhoid cases were detected in the PedaTyph recipients, compared with 11 cases in the placebo arm [54]. Another study to assess the vaccine immunogenicity was conducted in 400 children aged 3 months to 5 years, randomized to receive either 1 or 2 doses of PedaTyph. After 1 vaccine dose, anti-Vi antibody titers increased 9-fold (to 2.08 μg/mL from a baseline of 0.22 μg/mL); further, 8 weeks after vaccination, 83% of the children showed seroconversion (≥4-fold increase over preimmunization anti-Vi antibody levels) [55]. A subset of participants (40 vaccinated children) who had received PedaTyph (1 or 2 doses) was recalled at 30 months to assess the longevity of the immune response. Another 10 children who had not received a typhoid vaccine were also included as controls. Antibody titers for participants who had received a single dose or 2 doses and for the control group were 14 (4.8–29.8) μg/mL, 17 (7.4–33) μg/mL, and 6.4 (.8–12) μg/mL, respectively. The children in the 2-dose group had higher antibody titers as compared to the single-dose group, although the difference was not significant [56, 57]. Currently, PedaTyph is available in the private market in India.

### Typbar Typhoid Conjugate Vaccine From Bharat Biotech International Limited, India

This is the only WHO-prequalified TCV on the market. Typbar TCV is formulated by conjugation of Vi-polysaccharide to tetanus toxoid. In an initial Phase IIa/IIb study, safety and immunogenicity were evaluated in adolescents 13–17 years of age and children 2–12 years of age. Single and 2 doses of 25 μg/0.5mL and 2 doses of 15 μg/0.5mL were tested. This study was followed by a large Phase III study using a single 25 μg dose. The study was open-label in participants 6 months to 2 years of age and was randomized and double blinded with Vi-polysaccharide vaccine as the comparator in participants 2–45 years of age. The vaccine was well tolerated. At 6 weeks postvaccination, a 4-fold rise in anti-Vi antibody titers compared to baseline was seen in 98% of participants aged 6 months to 2 years, and 97.3% of participants aged 2–45 years. Compared to the Vi-polysaccharide vaccine recipients, anti-Vi antibody titers were more than 3 times higher among Typbar TCV recipients. At 2 years postvaccination, anti-Vi antibody titers remained higher in Typbar TCV recipients, compared to Vi-polysaccharide vaccine recipients (GMTs of 82 vs 46, respectively). A subset of participants from all age groups in the study received a booster dose 2 years after primary immunization. At 6 weeks postboost, all age groups showed a strong booster response, and postboost titers were higher than at 6 weeks after primary immunization with Typbar TCV [58].

The efficacy and immunogenicity of Typbar TCV was evaluated and compared to the Vi-polysaccharide vaccine in a human challenge study at the University of Oxford. Using the primary endpoint of bacteremia or fever, the vaccine efficacy of Typbar TCV was found to be 54.6%, compared with 52% for the Vi-polysaccharide vaccine. A post hoc analysis of the study data using alternative diagnostic criteria, such as would be clinically relevant (ie, fever of 38^°^C or higher preceding *S.* typhi bacteremia confirmed by blood culture), showed vaccine efficacy estimates of 87.1% for Vi-TT and 52.3% (95% CI, −4.2 to 78.2) for the Vi polysaccharide vaccine. The seroconversion rate was 100% for Typbar TCV and 88.6% for Vi-polysaccharide vaccine recipients [59]. This study showed clinical protection conferred by a TCV. However, since the study was conducted in largely naive British adults, the extrapolation of these results to children in endemic countries needs further evaluation in endemic settings.

Typbar TCV is licensed as a single-dose vaccine and currently registered in 4 countries (India, Nepal, Cambodia, and Nigeria). Typbar TCV was WHO-prequalified in January 2018, allowing organizations such as United Nations International Children’s Emergency Fund, Gavi, the vaccine alliance, and Pan American Health Organization to procure the vaccine for public health vaccination programs across the world [18].

As per the October 2017 meeting of the Strategic Advisory Group of Experts on Immunization, SAGE recommended the introduction of a TCV for infants and children over 6 months of age as a single dose in typhoid endemic countries. The introduction of TCV should first be prioritized in countries with the highest burden of disease or a high burden of antimicrobial-resistant *S.* typhi. SAGE also recommended catch-up vaccinations wherever feasible, with priority for catch-up in the youngest age groups (up to 15 years of age), depending on local epidemiology [12].

### Typhoid Vi Capsular Polysaccharide Tetanus Toxoid Conjugate Vaccine of M/s Cadila Healthcare Limited

Vi Capsular Polysaccharide Tetanus Toxoid Conjugate Vaccine (ZyVac-TCV), manufactured by Cadila Healthcare Limited, India, is the most recently licensed TCV. A Phase II/III study to demonstrate the noninferiority of ZyVac-TCV to Typbar TCV in healthy individuals aged 6 months to 45 years was initiated in 2016 [60]. A total of 117 participants (58 adults 18–45 years of age and 59 children 6 months to 17 years of age) out of 119 in the ZyVac-TCV arm completed the study, and 119 out of 121 participants (60 adults, 59 children) in the Typbar TCV arm completed the study. The seroconversion rate among ZyVac-TCV recipients was 94.8% (96.6% in adults and 93.1% in children), compared with 91.6% (91.7% in adults and 91.5% in children) for Typbar TCV recipients. The GMT of anti-Vi antibodies among ZyVac-TCV recipients was 1121 EU/mL (adults, 1411; children, 891.1), compared with 1104 EU/mL (adults, 1199; children, 1014) among Typbar TCV recipients. ZyVac-TCV was deemed noninferior to Typbar TCV and received marketing authorization in India in 2017 [61].

## NEXT-GENERATION VACCINES

### Protein-based Subunit Vaccine, Generalized Modules of Membrane Antigens, and Protein Capsular Matrix Vaccine

As an alternative to TCVs, the use of recombinant or purified proteins as subunit vaccines has also been pursued. These subunit vaccines can potentially elicit both T-cell and antibody responses. To date, outer membrane protein and adhesin have been evaluated as subunit vaccine candidates in animal studies [62]. Another approach being considered is that of Generalized Modules of Membrane Antigens (GMMA). GMMA are small, 50 to 90 nm diameter blebs shed from the surface of Gram-negative bacteria [63]. The deletion of proteins that span the periplasm of a bacterial membrane, as well as some other genes, enhances the shedding of GMMA. GMMA can deliver both surface polysaccharide and outer membrane proteins to the immune system. The GMMA production process is simple and high yield.

Another approach similar to the conjugation of polysaccharide is the Protein Capsular Matrix Vaccine (PCMV) technology developed by John Mekalanos’ group at the Harvard Medical School. PCMV consists of polysaccharide or capsular antigens that are entrapped in a cross-linked protein matrix with minimal or no covalent linkage of the capsular antigen to the protein. PCMV stimulate the immune system in a manner similar to glycoconjugates and offer a low-cost alternative for vaccine development against capsulated microorganisms [64].

### Multivalent Vaccine Concepts

In recent years, the burden of *Salmonella* paratyphi along with *S.* typhi is becoming more evident, particularly in Asia, and this has increased interest in *S.* typhi–paratyphi A bivalent vaccines [65]. The global disease burden studies often combine the typhoid and paratyphoid disease burdens [66], while many community-based studies estimate the *S.* paratyphi incidence separately. A pooled estimate of community-based studies in India suggested a paratyphoid incidence of 105 (74–148) per 100 000 person years, indicating its importance [9]. Several bivalent *S.* typhi and *S.* paratyphi A vaccine candidates are in preclinical development.

In addition, the burden of invasive nontyphoidal *Salmonella* (iNTS) disease, caused by *Salmonella enterica* serovar Typhimurium and *Salmonella enterica* serovar Enteritidis, is a serious public health concern in sub-Saharan Africa. Recent studies estimate that there are 600 000 to 3.4 million cases of iNTS disease every year, more than half of which occur in sub-Saharan Africa [67]. Antimicrobial resistance is also common, leading to the increased cost and duration of iNTS disease treatment. Hence, a trivalent vaccine (*S*. typhi, *S.* Enteritidis, and *S*. Typhimurium) is a logical step forward for control of enteric fever cases in sub-Saharan Africa.

## WAY FORWARD

The major burden of typhoid fever is borne by preschool and school-aged children [5, 16, 17] with increasing evidence of a significant burden in children <2 years of age [21]. There is therefore an urgent need for the deployment of improved typhoid vaccines that can be effective in younger children and provide longer durations of protection. The recent WHO prequalification of Typbar TCV is an important first step. The WHO released a position paper in March 2018 calling for the integration of a prequalified TCV into routine immunization, along with catch-up campaigns for children up to 15 years of age. Additional TCVs will be prequalified by the WHO in the coming years. The presence of a Gavi mechanism for TCV implementation and funding for TCV efficacy and effectiveness studies will promote TCV-based typhoid control programs in endemic countries, incorporating TCV into the national immunization programs of endemic countries.

A lack of data showing the clinical efficacy or effectiveness of TCVs in children residing in resource-limited settings is a major knowledge gap, though few studies have demonstrated the clinical protection provided by TCVs [41, 54]. With recent funding from the Bill & Melinda Gates Foundation and the European and Developing Countries Clinical Trials Partnership [68] for TCV efficacy and demonstration studies, data on clinical efficacy, effectiveness, and health economics are expected in the coming years. Many countries have limited information on disease burdens and disease locations due to the lack of diagnostics and surveillance systems. Without this information, it is difficult to prioritize typhoid vaccination relative to other health investments and to make decisions regarding age or geographic target populations for introduction. The ongoing effectiveness studies (Bangladesh, Nepal, Malawi, Burkina Faso, Ghana, and Democratic Republic of Congo) will be critical for generating much-needed effectiveness data using case-control or cohort study designs.

Since typhoid is endemic in resource-poor countries, the greatest demand for vaccines is expected primarily from the public market, where financial incentives for the vaccine manufacturer remain limited. It will be a challenge to keep manufacturers interested in TCV production and even more difficult to drive development interest for bivalent typhi–paratyphi A and trivalent iNTS disease vaccine candidates. In order to most effectively engage manufacturers, it may be critical for stakeholders and funding agencies to make market commitments on demand to offset commercial development risks.

## CONCLUSIONS

Typhoid vaccines have evolved over time to adapt to public health and programmatic needs. Efforts should be continued to address barriers and improve the uptake of current vaccines to control typhoid fever and to help curb the emergence of drug resistance. With a recent commitment from Gavi to support the introduction of TCV in eligible countries and increased funding from several stakeholders for additional typhoid studies, momentum appears to be in favor of wider TCV use.

## Notes

***Supplement sponsorship.*** This supplement is funded with support from the Coalition against Typhoid Secretariat, housed at the Sabin Vaccine Institute in Washington, DC and made possible by a grant from the Bill & Melinda Gates Foundation.

***Potential conflicts of interest.*** T. S., H. C., T. A. W., V. M., J. L., J. H. K., J.-L. E., and S. S. are employed by the International Vaccine Institute and are involved in SK Bioscience (South Korea) and Biofarma (Indonesia) Vi-DT vaccine development projects. All other authors report no potential conflicts. All authors have submitted the ICMJE Form for Disclosure of Potential Conflicts of Interest. Conflicts that the editors consider relevant to the content of the manuscript have been disclosed.
